# Utilization of Solid Waste as a Substrate for Production of Oil from Oleaginous Microorganisms

**DOI:** 10.1155/2018/1578720

**Published:** 2018-05-16

**Authors:** Fortunate Laker, Arnold Agaba, Andrew Akatukunda, Robert Gazet, Joshua Barasa, Sarah Nanyonga, Deborah Wendiro, Alex Paul Wacoo

**Affiliations:** ^1^Department of Chemistry, Faculty of Science, Kyambogo University, P.O. Box 1, Kyambogo, Uganda; ^2^Department of Microbiology and Biotechnology Centre, Product Development Directory, Uganda Industrial Research Institute, P.O. Box 7086, Kampala, Uganda; ^3^Yoba for Life Foundation, Hunzestraat 133-A, 1079 WB Amsterdam, Netherlands; ^4^Department of Molecular Cell Physiology, VU University Amsterdam, De Boelelaan 1085, 1081 HV Amsterdam, Netherlands; ^5^Department of Nursing, Muni University, P.O. Box 725, Arua, Uganda

## Abstract

The overwhelming demand of oil and fats to meet the ever increasing needs for biofuel, cosmetics production, and other industrial purposes has enhanced a number of innovations in this industry. One such innovation is the use of microorganisms as alternative sources of oil and fats. Organic solid waste that is causing a big challenge of disposal worldwide is biodegradable and can be utilized as substrate for alternative oil production. The study evaluated the potential of isolated yeast-like colonies to grow and accumulate oil by using organic solid waste as substrate. Of the 25 yeast-like colonies isolated from the soil samples collected from three different suburbs in Kampala district, Uganda, 20 were screened positive for accumulation of lipid but only 2 were oleaginous. The NHC isolate with the best oil accumulation potential of 48.8% was used in the central composite design (CCD) experiments. The CCD experimental results revealed a maximum oil yield of 61.5% from 1.25 g/L cell biomass at 10 g/L of solid waste and temperature of 25°C. The study revealed that organic solid waste could be used as a substrate for microbial oil production.

## 1. Background

Vegetable oils are the most traded, with palm oil being the most preferred due to its wide application in food, cosmetics, and biofuel industries. As the high demand of vegetable oil is being met, on the other hand, there is depletion of natural resources, a rise in poor cultivation practices, and eventually altered global climatic conditions that pose a threat to food security [1]. This therefore calls for exploration into other possible sources of oil production. Microorganisms such as oleaginous yeast have been found to accumulate large quantities of oil and can be utilized as alternative sources in oil production [2]. The composition of oleaginous yeast oil does not greatly differ from that of the vegetable oil, since their fatty acid compositions are comparable [3]. In addition, the microorganisms have a high growth rate and require minimal land space, and their oil production is not affected by climatic changes; moreover, they do not cause depletion of natural resources and hence do not pose a threat to food security [4]. However, the major challenge is the high cost of production [5]. Solid waste, whose disposal is a global challenge (ref.), can be effectively utilized as a cheaper substrate for alternative oil production.

In Kampala, the capital city of Uganda, approximately 28,000 tons of solid waste are collected monthly, of which about 90% are organic [6]. Management of the waste is continuously challenged by the rapidly increasing population of residents, increase in the rate of economic activities, and lack of sufficient funding from central government [7]. The waste is characterized by ≥13.5 g/Kg dry weight of nitrogen, ≥2 g/Kg dry weight of phosphorus, ≥6 g/Kg dry weight of potassium, and a very high carbon content [6]; it can be efficiently used as a substrate for microbial oil production. There is, therefore, a need to screen for the best functional oil-producing microorganisms and determine the optimal conditions. In this study, possibility of producing microbial oil from oleaginous yeast using solid organic waste as a substrate was investigated.

## 2. Materials and Methods

### 2.1. Soil Sample Collection

Seven soil samples were collected from wastes dumping sites in Kyambogo University, Banda, and Kireka, suburbs within Kampala District, Uganda. The samples were obtained approximately 2–8 cm below the soil surface and were stored in sterile transparent polythene bags at room temperature prior to transfer to the laboratory.

### 2.2. Isolation of Yeast Cells

The yeast cells were isolated on potato dextrose agar (PDA) (Conda, Madrid, Spain) plates which contained dextrose 20.0 g/L, infusion from potato 200.0 g/L, bacteriological agar 15.0 g/L, and 50.0 mg chloramphenicol supplement. The supplement was initially dissolved in 3.0 mL of absolute ethanol prior to being added to the medium base. The soil samples (5.0 g) were suspended in 9.0 mL of sterilized deionized water. The suspensions were thoroughly mixed for one minute at 2600 rpm using a vortex mixer (Stuart, Florida, USA) and subsequently followed by a tenfold serial dilution using sterile-buffered peptone water broth (Conda, Madrid, Spain). From each sample suspension, a dilution from 10^−4^ to 10^−7^ was chosen and an aliquot of 0.3 mL from each was spread onto PDA. The plates were incubated (ESCO Isothermal Incubator, Singapore) at 30°C for a period of 3 days. Yeast-like colonies were isolated and subsequently cross-streaked onto fresh PDA plates.

### 2.3. Screening for Oleaginous Yeasts

A loop full of each isolate suspension was inoculated into 100 mL nitrogen limiting medium contained in 250 Erlenmeyer flask. The medium contained the following in g/L: glucose 70, (NH_4_)_2_SO_4_ 0.1, KH_2_PO_4_ 0.4, MgSO_4_·7H_2_O 1.5, ZnSO_4_ 0.0043, CaCl_2_ 0.003, MnCl_2_ 0.0012, CuSO_4_ 0.0005, and yeast extract 0.795. The samples were incubated in an incubator shaker (Excella E25, New Brunswick Scientific, USA) at 150 rpm and 30°C for 4 days. The samples were then analyzed for their cell dry weight and percentage lipid content as described by Leasing [8]. The screening of samples was carried out in triplicate.

### 2.4. Characterization of Microbial Oil

The oil was structurally characterized by a Fourier transform infrared spectrophotometer (FTIR) (Perkin Elmer, Massachusetts, USA) as described by Wacoo et al. [9], with slight modification. Briefly, a thin layer of the microbial oil sample was placed in between the potassium iodide cells and held firmly by the cell-holder prior to being placed into the FTIR for read-up. The spectrum for the microbial oil was measured from 400 cm^−1^ to 4000 cm^−1^ at a scan speed of 500 nm/min. The spectrum for the microbial oil was plotted on the same axis as those of sunflower, coconut, and palm oil for comparative purpose.

### 2.5. Solid Waste

#### 2.5.1. Collection and Treatment

The solid waste for the production of microbial oil was collected without sorting waste disposal sites of a market, a residential place, and a restaurant. The waste was sorted and the organic solid waste was sun-dried and subsequently ground into a powder prior to being used in the production experiment. The powder waste was used as a substitute of glucose in the nitrogen-limiting medium mentioned in Section 2.3.

#### 2.5.2. Determination of the Glucose Equivalent from the Solid Waste

The sorted waste was first hydrolyzed using concentrated sulphuric acid followed by a method described by Saeman et al. [10]. Estimation of glucose from the hydrolyzed waste was done according to Miller [11].

#### 2.5.3. Central Composite Design Optimization of Cultivation Conditions for Microbial Oil Production

Central composite design was used to efficiently identify the optimum values of the temperature and solid waste which could lead to high oil accumulation. A two-factor five-level central composite design was therefore used to study the effect of solid waste and temperature on the oil yield of the selected yeast. Thirteen experiments were sufficiently used to estimate the second-order regression coefficients for the two variables as shown in (1) and the levels of the independent variables as shown in Table 1. The predicted values were determined by a model fitting technique using the design expert software. Regression analysis was performed on the experimental data and evaluation for significance of fit of the model done as required.

(1) Microbial  oil  yield %=a0+a1X1+a2X2+a12X1X2+a11X12+a22X22.

*Equation (1)*. Second-order polynomial model equation is used to express yield as a function of independent variables solid waste composition and temperature, where *a*0 is the interception coefficient, *X*_1_ and *X*_2_ are amount of solid waste and temperature, respectively, *a*12 is the interaction coefficient, and *a*11 and *a*22 are the quadratic terms.

## 3. Results and Discussion

### 3.1. Isolation of Oleaginous Yeasts

In the current study, 25 oleaginous microorganisms with yeast-like colonies were isolated on PDA containing chloramphenicol antibiotics (Table 2). Only three yeast-like colonies were isolated from the soil samples from Banda and Kireka compared to 22 from Kyambogo University.

### 3.2. Screening for the Oleaginous Yeasts

All the isolated yeast-like colonies were screened for accumulation of lipid as described in Section 2.3. The results revealed that all the isolates could utilize the nitrogen-limiting media for their growth. The cell biomass varied from 1.27 g/L to 9.49 g/L for isolates NHC and GC2N, respectively. The potential of the isolated yeasts to accumulate lipid varied greatly. Out of the 25 isolates, 20 were positive for accumulation of lipid but only 2 isolates (Table 2) were defined as oleaginous yeast with lipid accumulation of more than 20% of cellular dry weight [12]. Sample NHC with the lowest cell biomass of 1.27 g/L accumulated the highest lipid content of 48.8% and was thus selected for further studies.

### 3.3. Characterization of the Microbial Oil Using FTIR

**Figure 1:**
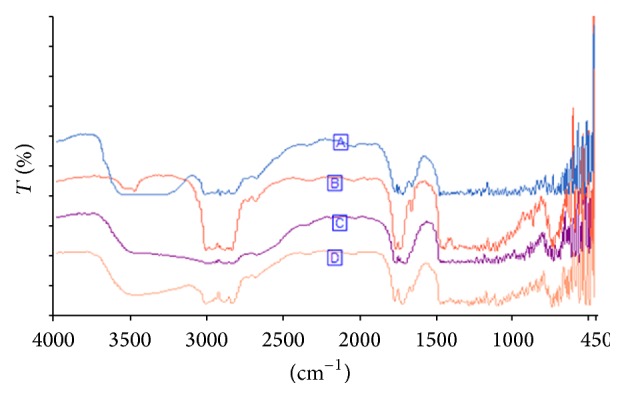
FTIR spectra of (A) sunflower oil, (B) refine coconut oil, (C) crude palm oil, and (D) oil extracted from yeast (current study).

The microbial oil from NHC isolate was characterized as described in Section 2.4. The spectrum from the microbial oil was plotted on the same axis as those of sunflower, coconut, and palm oil as shown in Figure 1. Compared to the three vegetable oils named above, the functional group of yeast oil was identified using the FTIR. The absorption peaks for functional group (carbonyl ester group) appeared at position from 1650 to 1850 cm^−1^. Many other researchers identified carbonyl ester groups at this position [13–15]. The peaks corresponding to the C-H stretching vibrations appeared ranging from 2920 cm^−1^ to 3010 cm^−1^.

The microbial oil was quite similar to coconut and sunflower oil at position 1655 cm^−1^ and this peak was attributed to* cis* -C=C- stretch [16]. This therefore indicates that, like refined coconut and sunflower oil, microbial oil contained some unsaturated fatty acids. Previous studies reported approximately 91% unsaturated fatty acid in sunflower oil [3]. Although crude coconut oil has been reported to contain >91% saturated fatty acid [17], the current study used refined coconut oil and it revealed that it contained fairly high content of unsaturated fatty acid as depicted at peak 1655 cm^−1^ (Figure 1  (B)). Microbial oil had approximately equal stretch at this position similar to the refined coconut oil. However, microbial oil was quite different from crude palm oil, which did not have stretch at this position.

### 3.4. Solid Waste

#### 3.4.1. Determination of Glucose Equivalent from the Solid Waste

**Figure 2:**
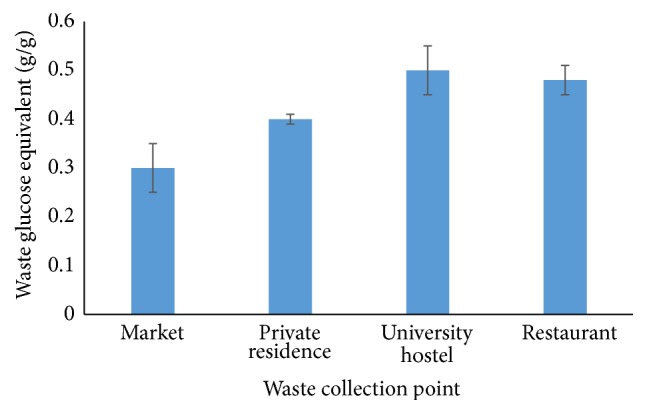
Characterization of solid waste collected for microbial oil production.

Glucose has been reported as the best substrate for microbial oil production, since it can be assimilated by most oleaginous microorganisms to produce oil [18]. Therefore, equivalent glucose contents from each collected waste were determined as described by Saeman et al. [10] and Miller [11]. The results of equivalent glucose from the collected solid waste are shown in Figure 2. The equivalent glucose contents per gram of waste were 0.5 g, 0.48 g, 0.4 g, and 0.3 g for wastes collected from the university hostel, restaurant, home, and market, respectively. The high glucose content in the wastes from the university hostel may be attributed to the high content of digestible food remains and papers, whereas the low glucose content in the market wastes may be due to decomposing food waste and indigestible solid materials.

#### 3.4.2. Central Composite Design Optimization of Cultivation Conditions for Microbial Oil Production

The corresponding results and the predicted values from the CCD are shown in Table 3. For the optimization purposes, the amount of solid waste and temperature were selected as the independent variables. The generated data from the CCD were analyzed by multiple regressions using design expert software to fit the quadratic polynomial model.

As shown in Table 3, the amount of solid waste and temperature had significant effects on the percentage of oil yield attained. Considering the results from all the runs, the concentration of microbial biomass stretched from 0.55 g/L to 1.25 g/L (data not shown) and the content of microbial oil ranged from 21.15% to 61.5%. However, the trend in yield was neither constant nor uniform; that is, increase in temperature and/or glucose concentrations did not result in an increase in the yield. This may have been brought about by incomplete extraction of cells from the reactor and centrifuge tubes among others. Comparative analysis between both actual and predicted yields indicated that there was a very high correlation between them, giving a very small deviation. Comparative analysis of the microbial biomass to previous studies indicated that the yield was quite low, although the oil contents were highly similar [19].

(2) Yield=21.13−8.08X1−10.28X2+6.33X1X2+7.94X12+9.36X22.

*Equation (2)*. This is the final equation in terms of coded factors.

Equation (2) shows the values of coefficient and regression models which correctly described the experimental data. The regression model indicated correlation between the two variables (amount of solid waste and temperature) that were used for microbial oil production with correlation coefficient (*R*^2^) of 0.98. Therefore, the value of this *R*^2^ strongly suggested that the experimental models represent the relationship between the experimental results and the predicated values. The predicted values were sufficiently correlated with the actual values as shown in Table 3.

The correlation obtained from the regression analysis (Table 4) was very positive and significant. It could be observed that the fit was almost 100% as lack of fit was almost negligible (<0.0001) and hence statistically significant. The analysis of variance (ANOVA) showed that the model can predict the interactions of the independent variable with 95% confidence limit.

**Figure 3:**
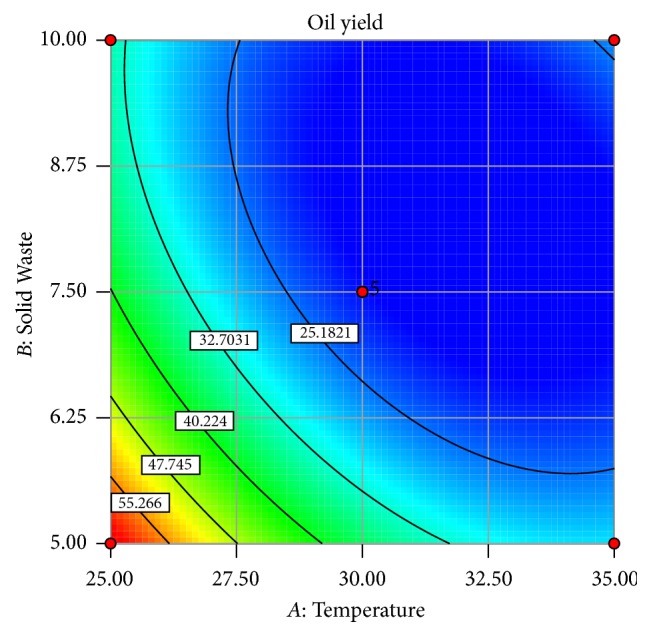
The graph of the predicted response developed as a function of solid waste concentration and temperature.

The predictability plot as a function of the amount of solid waste and temperature is shown in Figure 3. The oil yield increased quadratically with decrease in both glucose concentration and temperature. The best oil percentage yield was achieved at the lowest temperature of 25°C and the lowest amount of solid waste of 5 g/L. The predicted results obtained were very close to the expected values.

## 4. Conclusion

This study has not only demonstrated that oleaginous microorganism can be programmed for maximum cell biomass generation and oil production but also revealed the ability of the oil-producing microorganism to turn nuisance organic solid waste into valuable oil. Utilization of solid waste as a substrate for production of oil by oleaginous microorganism is a novel process that establishes the economic value of wastes besides proving a solution to the waste disposal challenge.

## Figures and Tables

**Table 1:** Levels of independent variables.

Independent variables	Lower level	Upper level
Solid waste (g)	5	10
Temperature (°C)	25	35

**Table 2:** Isolation and screening for lipid-producing yeasts.

Sampling place	Isolate	Cell dry weight (g/L)	Lipid accumulation (%)
Kyambogo University	GC2A	2.94	5.8
	GC2B	7.0	3.3
	GC2C	2.23	3.6
	GC2D	7.03	8.1
	GC2E	2.57	2.7
	GC2F	2.66	0.4
	GC2G	3.21	3.4
	GC2H	1.64	7.3
	GC2J	2.24	8.0
	GC2K	3.58	22.6
	GC2M	2.18	11.5
	GC2N	9.49	0.4
	GC1A	4.85	0.0
	NHA	5.80	0.0
	NHB	3.57	0.0
	NHC	1.27	48.8
	NHD	5.11	6.3
	NHE	5.40	11.1
	NHF	6.04	4.8
	NHG	2.98	7.0
	NHH	6.69	0.0
	NHJ	1.57	0.0
	NHK	4.77	11.9

Banda	BCCA	7.21	5.5
	BCCB	2.22	16.2

Kireka	KA	2.41	11.6

All the results are means ± standard deviation of triplicate analysis.

**Table 3:** Central composite design and the corresponding experimental results and predicted values.

Runs	Factors		Microbial oil yield	
	Solid waste [*X*_1_]	Temperature [*X*_2_]	Actual (%)	Predicted (%)
1	7.5	30	21.15	21.29
2	7.5	22.9289	53.2	54.38
3	7.5	30	21.4	21.29
4	5	25	61.5	62.79
5	7.5	30	20.8	21.29
6	5	35	28.3	29.75
7	3.96447	30	50.7	48.43
8	7.5	30	21.11	21.29
9	10	35	28	26.24
10	7.5	30	22.05	21.29
11	7.5	37.0711	24.6	25.32
12	10	25	35.9	34.3
13	11.0355	30	23.3	25.57

**Table 4:** ANOVA statistical results for the response surface quadratic model.

Source	Sum of squares	df	Mean square	*F* value	*p* value prob. > *F*	
Model	2396.26	5	479.25	152.12	<0.0001	Significant
*A*: temperature	831.23	1	831.23	263.84	<0.0001	
*B*: solid waste	522.44	1	522.44	165.83	<0.0001	
*AB*	160.02	1	160.02	50.79	0.0002	
*A* ^2^	554.22	1	554.22	175.91	<0.0001	
*B* ^2^	442.52	1	442.52	140.46	<0.0001	
Residual	22.05	7	3.15			
Lack of fit	21.14	3	7.05	30.96	0.0032	Significant
Pure error	0.91	4	0.23			
Cor. total	2418.32	2				
